# Adaptation of PCR-based library preparation for MGI platform for cancer mutation testing in clinical setting

**DOI:** 10.1371/journal.pone.0323685

**Published:** 2025-06-04

**Authors:** P.A. Shatalov, M.P. Raygorodskaya, A.P. Shinkarkina, A.A. Traspov, A.V. Murzaeva, Y.A. Doroshenko, I.A. Leuhina, V.A. Mileyko, M. V. Ivanov, T.V. Grigoreva, A. A. Lebedeva, E.M. Veselovsky, L.D. Belyaeva, E. V. Belova, A.D. Kaprin, P.V. Shegai

**Affiliations:** 1 National Medical Research Radiological Centre of the Ministry of Health of the Russian Federation, Obninsk, Russia; 2 OncoAtlas LLC, Moscow, Russia; 3 Sechenov First Moscow State Medical University, Moscow, Russia; 4 Koltzov Institute of Developmental Biology of the Russian Academy of Sciences, Moscow, Russia; 5 Lomonosov Moscow State University, Moscow, Russia; CNR, ITALY

## Abstract

MGI platforms hold promise to become a widespread instrument for various clinical next-generation sequencing applications, from whole genome sequencing to COVID-19 genotyping. However, in the clinical oncology setting it is still restricted to large panel sequencing limiting capacity for routine biomarker screening. In this article, we describe our experience of tailoring amplicon-based library construction for the MGI platform. Illumina compatible reagents served as a prototype in order to introduce platform specific adapters. Elaborated reagent kits were used for BRCA1/2 or 34 oncogenes testing both with whole blood and FFPE-derived DNA. Our data show that amplicon-based DNBSEQ-tailored library preparation demonstrates sufficient analytical efficiency in terms of coverage uniformity (average MAPD 1.08 and 1.19 for ABC plus and Atlas plus panels) and amplicon drop-out rate (ranging from 0.3% to 2.5%). Additionally, it shows efficiency in terms of single sample sensitivity, maintaining 99% sensitivity compared to 99% for the Illumina prototype. We show that it also outreaches expected diagnostic parameters of MGI exome sequencing (99% vs 95% for WES). Per-amplicon coverage of sticky-end libraries sequenced on Illumina and MGI were highly correlated demonstrating that the platform itself does not introduce any bias to amplicon coverage. Across three tested variations of library preparation protocol, discordances were related to ligation mix component composition and resulted in underrepresentation of GC-low and GC-high amplicons and low uniformity as a result. Overall, we outline the successful adaptation of PCR-based library preparation for MGI signifying the importance of tailoring component composition of reagent kit for uniform coverage.

## Introduction

In current clinical practice, targeted next-generation sequencing (NGS) is the most widely utilized approach to screening for alterations with potential diagnostic, prognostic or predictive significance in patients with cancer. Whole exome sequencing (WES) and large targeted panels (LTP) are oftentimes used for diagnosis of hereditary cancer syndromes and for comprehensive genomic profiling of tumors [1]. Both WES and LTP are typically hybridization capture-based methods [2–4]. However, in current clinical practice of molecular pathology the utility of this approach may be limited due to the following:

1) a shortage of material or a high degree of degradation may result in a quality control failure of a significant number of samples (up to 30% [5]). In this case, the patient will not receive genomic testing;
2) Hybridization capture is the most efficient for large targeted panels [6]; however, in routine clinical practice, only a handful of biomarkers are analyzed and can be interpreted according to the treatment guidelines. Therefore, the use of hybridization capture might not be cost-efficient, especially for routine diagnostics [7].

In addition to the indicated fundamental limitations of hybridization enrichment, a lack of simple and reliable for routine use laboratory protocols as well as associated obstacles with data analysis and interpretation may pose challenges to the widespread introduction of NGS into clinical practice [5]. From this point of view, enrichment methods based on multiplex PCR are significantly advantageous compared to NGS, since the working skills in real-time PCR are sufficient for the preparation of DNA libraries and troubleshooting. Various options for PCR-based enrichment and their application in oncology are listed in Table 1.

**Table 1:** Various options for PCR-based enrichment and their application in oncology.

Method (manufacturer)	Preanalytical steps	Examples of use in oncology	Advantages and limitations	Platforms for which approach have been tested	Reference
Ampliseq (Illumina, CA, USA)	1) multiplex PCR for the enrichment 2) Primer removal and end-repair 3) Adapter ligation 4) Library amplification with universal primers	1) Study of cancer-related genes (detection of somatic alterations) 2) Identification of molecular alterations for targeted therapy 3) Analysis of oncogenes and tumor suppressor genes	Advantages: 1) A relatively simple sample preparation protocol 2) Ability to use as little as 10 ng of DNA 3) Capability to analyze DNA from paraffin blocks (degraded DNA) 4) Rapid result turnaround 5) High accuracy and sensitivity of analysis Disadvantages: 1) Potential errors in the sequencing process 2) Not universally applicable across all platforms, adaptation required	1) Illumina 2) IonTorrent	[8]
Halo plex (Agilent Technologies, Inc, CA, USA)	Hybridization panel 1) Fragmentation of genomic DNA using restriction enzymes 2) Hybridization of fragmented DNA with Haloplex probes for targeted enrichment and sample indexing 3) Capture of target DNA 4) Ligation of captured, circularized fragments 5) Preparation of master mix, PCR amplification of target DNA 6) Wash of captured DNA 7) PCR amplification of target captured libraries 8) Purification of amplified, target libraries	Targeted gene sequencing for various diseases	Advantages: 1) Rapid result turnaround, protocol requires 6 hours 2) Capability to analyze 48–96 samples Disadvantages: 1) Requires 200 ng of DNA 2) Need for additional probe design before purchasing the kit 3) Requires multiple lengthy washes.	Illumina	[9]
Archer (ArcherDX, CO, USA)	Various protocols	1) Study of genes associated with cancer (detection of somatic variants) 2) Identification of gene variants for targeted therapy 3) Analysis of oncogenes and tumor suppressor genes 4) Fusions analysis	Advantages: 1) Approximately 10 ng of DNA is required. 2) Large volumes of data. 3) Facilitates the search for genes and variants associated with diseases, including in non-coding regions of the genome. Disadvantages: 1) High cost. 2) Labor- and time-consuming. 3) Involves more sample preparation steps, as compared to other methods. 4) Requires high sequencing coverage.	1) Illumina 2) IonTorrent	[10,11]
smMIP (multiple manufacturers)	Single cell 1) Attachment of specific probes to DNA 2) Denaturation followed by annealing 3) Hybridization with target DNA 4) Circularization	1) Non-invasive prenatal screening 2) diagnosis of oncological diseases and detection of disease recurrence, 3) evaluation of the effectiveness of drug treatment, tumor testing	Advantages: Single-cell sequencing can be performed on samples with suboptimal sample content. Disadvantages: Errors during PCR amplification.	Illumina	[12]

The widespread usage of MGI (MGI Tech, Inc.) sequencers based on DNA-nanoballs sequencing (DNBSEQ) technology raises the question of adaptation of conventional targeted sequencing methods to this platform. As compared to semiconductor sequencing used by Ion Torrent sequencers and bridge-PCR sequencing by synthesis methods used by Illumina sequencers, for MGI only hybrid-capture solutions are available [13–15].

The iterative adaptation process should include a comprehensive analysis of the quality of the resulting sequencing data and the ability to compare them with validated solutions. Among the technical characteristics that reflect the quality of sequencing, the standard ones include the uniformity of coverage across amplicons, coverage of the sequences close to the ends of the amplicons, where adapters are ligated, as well as contribution of fragment lengths and GC content to the representation of different target sequences [16–18]. In addition to the technical metrics, the ability to detect clinically significant genomic variants should be evaluated for all of the test systems introduced into clinical practice.

In this paper, the results of the adaptation of an amplicon-based NGS test-system for the MGI platform along with its direct comparison with WES and its original version on the Illumina platform will be discussed.

## Methods

### Sample collection

Various protocols, as well as the final versions Helicon ABC plus and Helicon Atlas plus, were tested on a total of 68 unique real-world samples (38 formalin fixed paraffin embedded (FFPE) blocks and 30 whole blood (WB) samples) from 56 patients referred for routine genetic testing. Data were accessed for research purposes for the purposes of this study between 20/11/2023 and 15/02/2024. The study received approval from the Ethics Committee at the National Medical Research Radiological Centre (Obninsk, Russia) and was conducted in compliance with the Declaration of Helsinki. Written informed consent was obtained from all patients. The patients underwent molecular genetic testing at the National Medical Research Radiological Centre. Authors had no access to information that could identify individual participants during or after data collection.

### DNA isolation and analyte quality control

DNA isolation followed established previously protocols [19]. Briefly, for WB samples, DNA isolation was performed using QIAamp DNA Blood Kits (Qiagen), while FFPE samples were processed using QIAamp DNA FFPE Tissue Kit (Qiagen). The concentration of the extracted DNA and prepared libraries was measured using the Fluo-200 fluorometer (AllSheng, China) with the Equalbit 1 × dsDNA HS Assay Kit (Vazyme, China) for quantifying double-stranded DNA. Quality control of the analyte included assessing the concentration of the extracted DNA (not less than 0.5 ng/μl) as well as the concentration of the DNA libraries (not less than 0.6 ng/μl, equivalent to 4 nmol for an average library length of 267 bp). In cases of low DNA concentration (ranging from 0.5 to 1.5 ng/μl), the number of PCR cycles was increased to 21. For low DNA library concentrations (ranging from 0.3 to 0.6 ng/μl), the feasibility of further sequencing was determined on a case-by-case basis, taking into account the DNA concentration, the percentage of tumor cells, and the extent of necrosis, as determined by the histopathological examination of the original sample.

### Illumina library preparation

The preparation of NGS libraries was carried out with enrichment of target regions through amplification using the Solo test ABC plus and Solo test Atlas plus (OncoAtlas, Russia), following the manufacturer’s instructions. Solo test ABC plus panel comprises 517 primer pairs (517 amplicons) within 6 cancer-related genes: BRCA1, BRCA2, ATM, CHEK2, PALB2, BRAF. Solo test Atlas plus panel comprises 474 primer pairs (amplicons) within 34 cancer-related genes: AKT1, AKT2, AKT3, ALK, ARAF, BRAF, EGFR, ERBB2, ERBB3, ERBB4, ESR1, FGFR1, FGFR2, FGFR3, FGFR4, H3F3A, HIST1H3B, HIST1H3C, HRAS, IDH1, IDH2, KIT, KRAS, MET, NRAS, PDGFRA, PIK3CA, PTEN, RAC1, RAF1, RIT1, ROS1, STK11, TP53.

### DNBSEQ adapted library preparation

**Fig 1:**
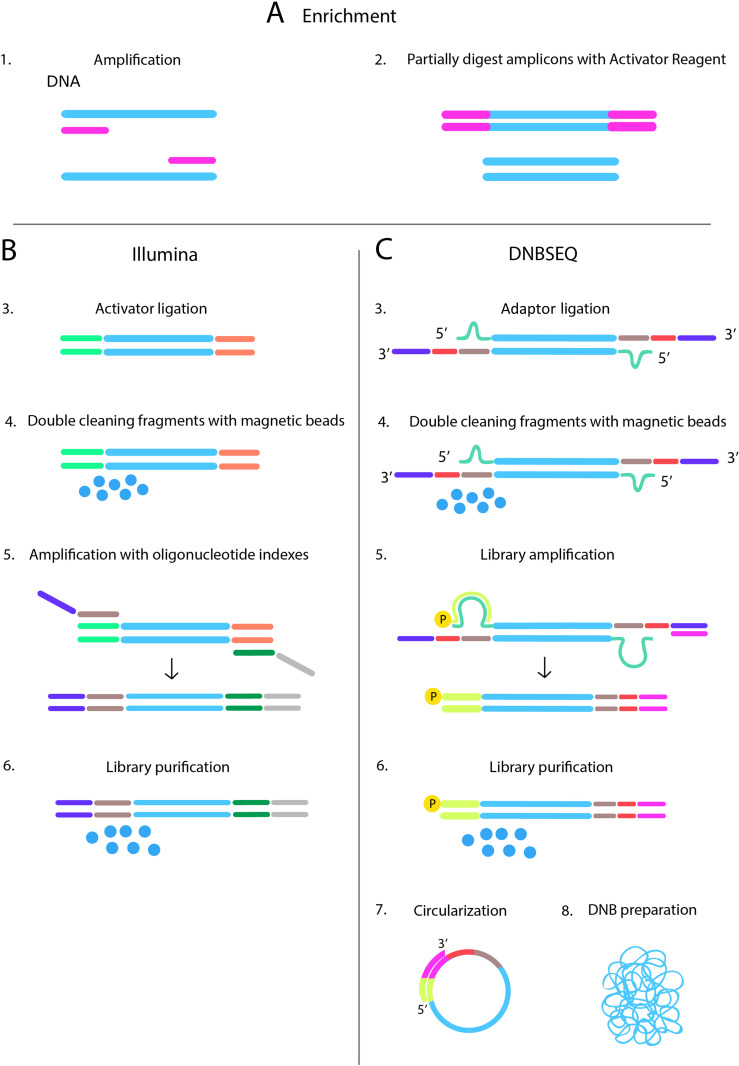
Steps in amplicon enrichment sample preparation with subsequent sequencing on the Illumina and MGI platform. A – common part, B and C – steps of the Illumina- and DNBSEQ-adapted protocols, respectively. DNB – DNA nanoballs.

DNBSEQ adapted library preparation protocol was proposed and consists of several key steps. In the first stage, DNA is enriched through multiplex PCR using two pools of uracil-modified primers (Fig 1A, step 1). The products of the first PCR reaction are combined and treated with an Activator (Fig 1A, step 2), a mixture of enzymes that cleaves the primers at uracil bases and generates sticky ends. During the ligation step (Fig 1A, step 3), barcoded adapters are ligated to the activator-treated amplicons via the sticky ends using ligase and ligation buffer. This is followed by a two-step purification of the libraries on magnetic beads (Fig 1A, step 4), which is necessary to remove residual genomic DNA, primers, and adapters. The resulting libraries are amplified using PCR Mix 2 and Primer Solution 3 (Fig 1A, step 5) and undergo additional purification on magnetic beads (Fig 1A, step 6). Finally, the libraries are circularized, and DNA nanoballs are created (Fig 1A, step 7–8) for subsequent sequencing.

The proposed protocol was initially tested and results were estimated preanalytically via DNA library concentration and fragment size estimation. Further testing and optimization of the DNBSEQ adapted protocol were focused on modifying the following key reagents, which are theoretically considered to have the greatest impact on DNA library and subsequent sequencing data quality: 5x PCR Mix 1 (components: Taq F Polymerase, KCl, Tricine, Tris(hydroxymethyl)aminomethane, Raffinose, Sucrose, Glycerol, Sorbitol, MgCl2, dNTP, Twin20, TMA with the variable components concentration across different tested protocols [OncoAtlas, Russia]), Activator (components: UDG and other unique components varying between protocols [OncoAtlas, Russia]), Ligase (OncoAtlas, Russia), and 5x Quick Ligation Reaction Buffer (LB002 [Evrogen, Russia]), 1x PCR Mix 2 (components: Potassium chloride, chemical purity, Tris aminomethane, Tricin, Raffinose-D(+), Sucrose, Glycerol, Sorbitol, Magnesium chloride, hexahydrate, dNTP, TWEEN20, Dimethyl sulfoxide, HS Taq DNA polymerase with the variable components concentration across different tested protocols [OncoAtlas, Russia]).

### Sequencing and data analysis

Pooled libraries were sequenced using either DNBSEQ-G50 (PE100) or Illumina NextSeq 500 (NextSeq control software v2.0.2/Real Time Analysis v2.4.11) with a 300 cycle NextSeq High Output Reagent Kit v2.5. FASTQ files were generated locally on the instrument for both platforms.

Reads were preprocessed for a removal of low-quality sequences (minimum mean read quality score 25) using the Prinseq-lite program [20]. Sequence data alignment to reference genome (GRCh37.p13) was performed with Burrows-Wheeler Aligner (BWA-mem, version 0.7.7-r441) [21]. Raw sequencing data quality was assessed with FastQC v0.11.7. To assess coverage uniformity of amplicon data MAPD was additionally calculated using in-house scripts as the median of the absolute values of log2 differences between the log2 of read count ratio values against the reference baseline for all adjacent amplicons in log2. Sequence logos were generated with Logomaker [22]. Sequenced fragment statistics was extracted from BAM files employing pybam package. Per-amplicon coverage statistics was assessed employing bedtools v2.26.0 [23]. Statistical analysis was performed using R, version 3.2.3.

### Whole exome sequencing

DNA was extracted from blood using QIAamp DNA kits. Libraries were prepared from 100–400 ng of DNA using the MGIEasy Universal DNA Library Prep Set (MGI Tech). DNA from FFPE samples was fragmented using S1 nuclease and USER enzyme mix. DNA and library quality were assessed using Qubit and Bioanalyzer 2100. Exome capture was performed using MGIEasy Exome Capture V5 Probe set. Sequencing was carried out on the DNBSEQ-G400 platform with 100x and 200x coverage for blood and tumor samples, respectively. For comparative analysis samtools was used to extract reads aligned versus regions covered by Solo test ABC plus and Solo test Atlas plus.

## Results

### DNBSEQ-adapted protocol development

The library preparation protocols for Illumina and MGI are different, with the key differences being the shapes of adapters and the indexing processes. DNBSEQ-adapted library preparation protocol schematically represented and compared to Illumina protocol on Fig 1 and described in Methods section. Briefly, the DNBSEQ library preparation protocol involves key steps: DNA enrichment via multiplex PCR with uracil-modified primers (Fig 1A, step 1), treatment with an Activator to generate sticky ends (Fig 1A, step 2), ligation of barcoded adapters (Fig 1A, step 3), two-step magnetic bead purification to remove contaminants (Fig 1A, step 4), amplification with PCR Mix 2 and Primer Solution 3 (Fig 1A, step 5), additional purification (Fig 1A, step 6), and circularization to create DNA nanoballs for sequencing (Fig 1A, step 7–8).

In this study, we developed four protocol variants (see Methods section) – DDD, III, IDI, and DDD-E – to investigate the impact of different reagent compositions on sequencing data quality. Each protocol name reflects the specific combination of key reagents used. The first letter corresponds to the type of PCR mix1 (D or I), the second to the ligase and ligase buffer (D or I), and the third to PCR mix2 (D or I). Additionally, the presence of an activator is indicated by the -E suffix. This systematic design allows for a structured analysis of how individual reagents influence sequencing performance.

**Fig 2:**
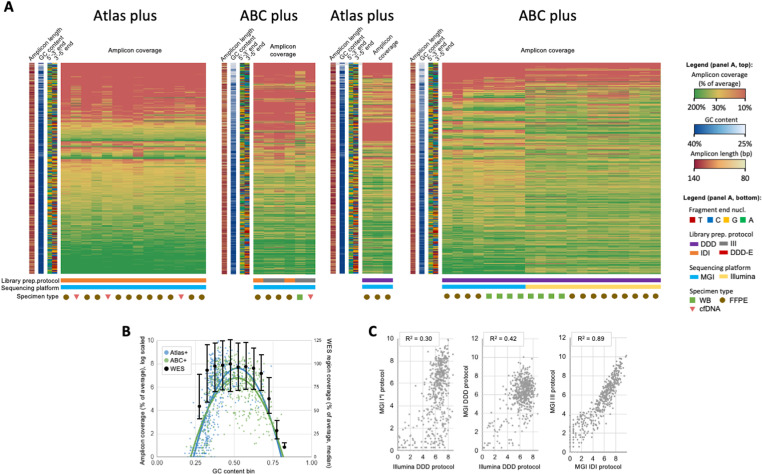
Analysis of biases in amplicon coverage across samples sequenced with diverse Ampliseq-based protocols (DDD/IDI/III – corresponds to variations of PCR mix1 (I/D), ligase/buffer (I/D) and PCR mix2 (I/D); ‘-E’ stands for alternative Digest Mix). **(A)** – heatmap of amplicon coverage (% of average coverage) across samples demonstrates significant variation in per-sample set of dropped out (10% of average sample coverage) amplicons depending on **i)** PCR mix 2 used **ii)** Digest Mix used. Coverage of amplicons by amplicon GC content analysis in samples sequenced on MGI platform (B) demonstrates extremely high amplitude between coverage of GC-low (30% and lower) and GC-moderate (40% and over) amplicons, while GC-high (70% and higher) amplicons demonstrate only slight decrease in coverage.(C) Per-amplicon coverage correlation between different protocols adopted for Illumina/MGI sequencing platforms.

The evaluation of different DNBSEQ-adapted protocol variants was conducted using small oligonucleotide primer panels, Solo test ABC plus and Solo test Atlas plus, comprising 517 and 474 amplicons, respectively. These panels are designed for molecular genetic studies in clinical oncology. The testing was performed on both tumor tissue (FFPE), whole blood samples (WB) and liquid biopsy samples (cfDNA), while sequencing was performed both on MGI and Illumina sequencing platforms. In total, 44 samples were analyzed (Fig 2A).

Following the series of experiments described below, the DDD protocol yielded the most favorable results. The results of protocol testing are presented in Fig 2A as a heatmap, where each strip corresponds to an amplicon, with green indicating higher amplicon coverage and red indicating lower amplicon coverage. Below the heatmap, a sequencing platform, sample type and the protocol tested can be seen. IDI and III protocols showed the worst results in terms of per-amplicon coverage uniformity (average MAPD 1.05, 1.71, 2.33 and 2.49 for DDD, DDD-E, IDI and III DNBSEQ-adapted protocol variants tested on WB samples employing ABC plus target panel respectively; and 1.31, 2.01, 2.45, 2.42 while tested on FFPE samples respectively). The comparison of poorly covered amplicons with the GC content of the insert demonstrated that the reason for the low uniformity of coverage may be GC-rich (>30%) and GC-poor (<30%) amplicons, whose coverage amounted to 5%, 3%, 20%, and 25% on average relative to the mean coverage in the sample across protocols III, IDI, DDD-E, and DDD with the probability of amplicon drop-out (relative per-amplicon coverage of 5% and lower) for amplicons with the corresponding GC content reaching 95%, 94%, 10% and 6% for different protocols respectively (Fig 2B). Replacing the second reagent (ligase and buffer: protocol variants IDI vs III) did not significantly affect protocol performance, including in comparison with Illumina, as seen by per-amplicon coverage Pearson correlation coefficient of 0.94 (Fig 2C).

The DDD protocol outperformed other protocol versions on both Illumina (ABC plus panel) and MGI sequencers (ABC plus and Atlas plus panels). On both platforms, the same sets of amplicons were underrepresented, indicating equivalent platform performance for the DDD version of the protocol (Fig 2A).

The amplicon coverage obtained with WES, ABC plus, and Atlas plus panels on the MGI platform, stratified by GC content, showed high dispersion of coverage of GC-low (≤30%) and GC-intermediate (40–60%) amplicons across all panels. Amplicon coverage of GC-low (≤30%) and GC-high (≥70%) amplicons was lower for ABC plus and Atlas plus panels compared to the matched regions from WES (Fig 2B).

Fig 2C illustrates the correlation between amplicon per-nucleotide coverage obtained with different library preparation protocols tested. The combined mean coverage obtained with the IDI and III protocols on the MGI platform showed a positive correlation of 0.58 with the DDD protocol on the Illumina platform. The correlation between the III and IDI protocols run on the MGI platform reached 0.94. The DDD protocol demonstrated a correlation of 0.65 between the two platforms.

Finally, all samples analyzed employing DDD protocol demonstrated 100% *in silico* sensitivity demonstrating superior efficacy of the protocol as compared to the III and IDI protocols (mann-whitney u test p-value < 0.05) applicable for clinical use.

Taken together, the DDD protocol outperformed the other protocol variations. Therefore, the DDD protocol was chosen for the following analysis.

### Performance of the DNBSEQ-adapted protocol

Next, we focused on a more detailed analysis of the technical and clinical efficiency of the DDD protocol variant (hereinafter referred to as the DNBSEQ-adapted protocol), which had previously outperformed other protocols. For this purpose DNBSEQ-adapted protocol was tested using Atlas plus and ABC plus amplicon panels. Atlas plus was used to sequence 15 FFPE samples, while ABC plus was used to sequence 14 samples, comprising 8 FFPE samples and 6 whole blood samples. The median total count of reads generated per sample was 5.9mln (range 2.3mln - 15.8mln). The average MAPD was 1.14 with a standard deviation of 0.13 for Atlas plus panel (1.19 ± 0.21 for pool 1, 1.14 ± 0.14 for pool 2) and 1.08 ± 0.33 for ABC plus panel (1.1 ± 0.35 for pool1 and 1.14 ± 0.28 for pool 2). Amplicon drop-out (defined as the relative per-amplicon coverage of 5% and lower) rate ranged from 0.3% to 1.3% for ABC plus panel and from 0.3% to 2.5% for Atlas plus panel.

**Fig 3:**
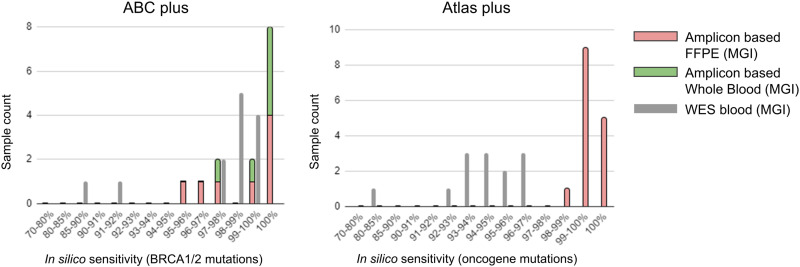
*In silico* sensitivity for the detection of pathogenic variants in the BRCA1/2 genes (ABC plus panel) and predictive biomarkers of ESMO Scale for Clinical Actionability of Molecular Targets (ESCAT) level I across major oncogenes (Atlas plus panel) was determined using EphaGen software. The analysis was conducted on datasets comprising 29 samples sequenced using the DNBSEQ-adapted protocol and compared with results of blood whole exome sequencing (WES) involving a total of 13 samples.

At first, we evaluated the sequencing data regarding their quality and clinical relevance of the DNBSEQ-adapted protocol. For this purpose *in silico* sensitivity was estimated using EphaGen software, utilizing the statistical framework described previously [18]. In essence, the read count covering each clinically relevant position, corresponding base quality, and mutant allele prior probability were assessed to predict the panel’s ability to detect the mutant allele at this position. Clinically relevant alterations were defined as pathogenic variants in the BRCA1 and BRCA2 genes for ABC plus panel, and predictive alterations of ESMO Scale for Clinical Actionability of Molecular Targets (ESCAT) level I, including common oncogenic variants in EGFR, KRAS, PIK3CA, and 31 other oncogenes for Atlas plus panel. For comparison, WES data from 13 whole blood samples obtained with a panel designed specifically for the MGI platform were utilized. The *in silico* sensitivity of WES was assessed using regions covered by the ABC plus and Atlas plus panels. As seen in Fig 3, both targeted panels demonstrated acceptable sensitivity surpassing WES sequencing for detecting clinically significant single nucleotide variants and small (<35 bp) indels (average 98.8% for ABC plus, 99.3% for Atlas plus, and 94.7% for WES). It should be noted that the lower *in silico* sensitivity of WES cannot be attributed to lower coverage, as EphaGen normalizes data according to coverage.

### DNA library characteristics

**Fig 4:**
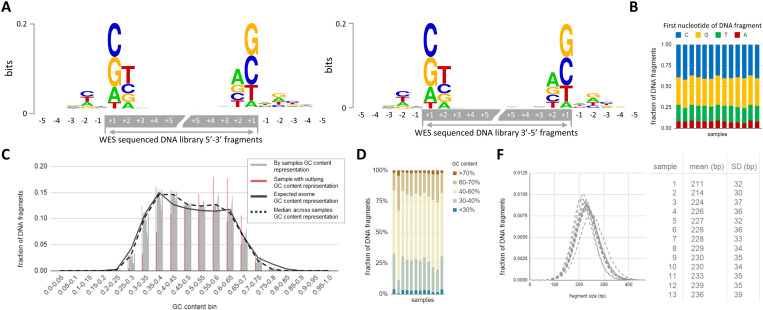
Characteristics of DNA libraries sequenced using MGI WES protocol. Sequence context of DNA fragment ends demonstrates equivalence of 5’-3’/3’-5’ DNA strands sequencing in terms of biased representation of fragments with terminal G or C nucleotides **(A)**. The bias was consistent across all samples **(B)** GC-content analysis of sequenced DNA fragments in comparison with expected by random sampling demonstrates slight overrepresentation of GC moderate (40-60%) regions at the expense of GC low (<30%)/high (>70%) (C) along with high variation in GC-low/moderate/high fragments representation across 13 samples **(D)**. Fragment distribution by length across 13 samples **(F)**.

**Fig 5:**
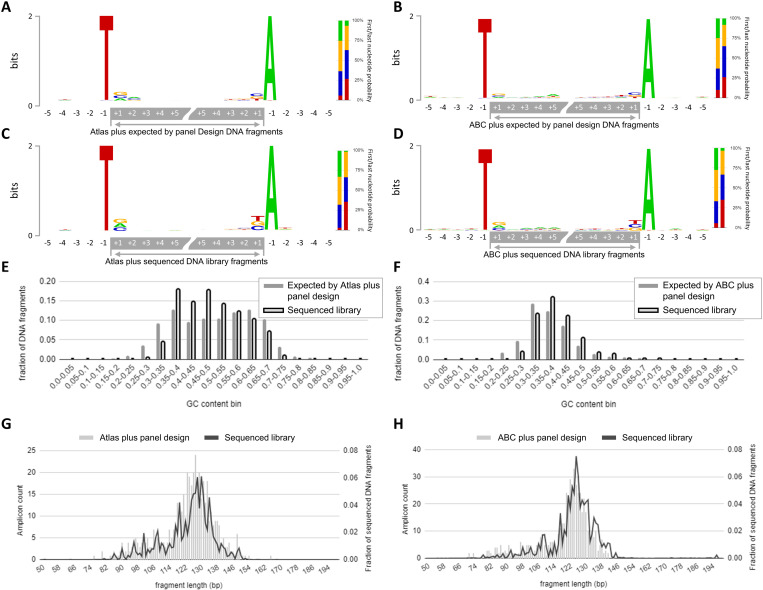
Characteristics of Atlas plus and ABC plus panel designs and DNA fragments sequenced using DNBSEQ-adapted protocol. Sequence context expected by panel (amplicon) design (A, B) and sequenced **(C, D)** DNA fragment ends demonstrates absence of nucleotide composition based bias in fragments representation **(B)**. GC content analysis of sequenced DNA fragments in comparison with expected by panel design (E, F) demonstrates significant overrepresentation of GC moderate (35-55%) amplicons at the expense of GC low (<30%)/high (>70%) amplicons. **(F)** – fragment representation by length across 29 samples.

To address the potential bias introduced by DNBSEQ-adapted protocol, DNA libraries were characterized using both WES and ABC plus/Atlas plus panels. WES was used as a reference since that panel was designed for the MGI platform. For this purpose, we examined the distribution of fragment lengths, the nucleotide composition at the ends of the sequenced fragments, and the GC-content of the fragments (Figs 4 and 5).

First, we analyzed the distribution of fragment lengths. The mean length in each sample ranged from 211 to 236 with a standard deviation ranging from 30 to 39. The distribution followed a normal pattern, with no bias detected (Fig 4F).

To assess the typical nucleotide content at the ends of fragments generated by WES of 13 WB samples and in the adjacent genomic regions we applied graphical representation with sequence logos. Guanine and cytosine were slightly overrepresented at the + 1 position (the terminal nucleotide of the fragment). No difference was observed between forward and reverse reads (per-nucleotide Fisher’s test, p-value > 0.05) (Fig 5A). The observed bias towards guanine and cytosine may be explained by the peculiarities of preparative DNA fragmentation, as breaks predominantly occur in CpG islands during sonication [24]. Terminal nucleotide content composition was similar between all 13 samples (ANOVA test, p-value > 0.05) (Fig 4B).

Next, we evaluated bias towards GC-content of whole fragments (Fig 4C and 4D). The expected GC-content of target regions exhibited a bimodal distribution (Fig 4C, solid line). The median GC content of sequenced fragments closely matched the calculated values, showing a trend towards sequences of intermediate GC-content (40–60%) at the expense of GC-low (<30%) and high (>70%) sequences (Fig 4C, dashed line). One sample exhibited a bias towards a higher GC level (57.4% higher within the range of 55–60% and 49% lower within the range of 35–40%) (Fig 4C, marked in red).

Thus, no significant biases were observed in the libraries generated for WES with the panel originally designed for MGI technology.

Next, we evaluated the parameters described above for libraries generated with the adapted protocol using the ABC plus and Atlas plus panels.

A comparison of fragment lengths specified in the panel design with the actual fragment lengths revealed no significant differences for either panels (Fig 5G and 5H).

The expected nucleotide composition of fragment ends and adjacent regions was calculated for each panel (Fig 5A and 5B) and compared with the actual composition (Fig 5C and 5D). Nucleotides immediately adjacent to the terminal nucleotides (−1 position) were exclusively A or T. This is explained by the peculiarities of the library preparation protocol, which involves the use of uracil-containing primers followed by excision with uracil N-glycosylase. The expected and actual nucleotide content at fragment terminal positions (+1 position) were similar, demonstrating the absence of nucleotide composition bias in fragment representation (Fisher’s test p-value > 0.05) (double stacked charts in the right part of each figure: Fig 5A-D).

GC-content analysis revealed that both ABC plus and Atlas plus panels were significantly biased towards intermediate GC amplicons (35–55%) while GC-low (≤40%) and high (≥70%) amplicons were significantly underrepresented (Fig 5E,F).

The number of sequenced fragments with GC content of 0–35% amounted to 4.8%, which is lower than the expected 13.9% for the Atlas plus panel and 27.2% compared to the expected 42.1% for ABC plus panel (Fisher’s test p-value 0.00018). The number of fragments with GC content of 70–100% was 0.9% compared to the expected 4.3% for the Atlas plus panel.

## Discussion

In this study, we conducted a comprehensive evaluation of a newly developed DNBSEQ-adapted protocol, which involved the ligation of technical sequences to amplicons treated with an enzymatic mixture containing uracil N-glycosylase. To optimize the protocol, we systematically compared various modifications differing in their component composition. Furthermore, we performed a technical validation of the DNBSEQ-adapted protocol to assess its technical robustness and clinical efficacy. Our findings demonstrate that this protocol offers significant improvements in performance, making it a promising approach for applications requiring high precision and reliability.

Adaptation of PCR-based enrichment for MGI requires consideration of several aspects associated with this approach (Fig 1). First, it is also crucial to ensure the circularization of each target amplicon, and this imposes restrictions on the size of amplicons and their diversity in a multiplex panel. Second, the difference in the GC-content and the “PCR-ableness” of the amplicons may lead to uneven amplification during the process of nanoballs production, which may lead to a critical decrease in the coverage of some loci. As an alternative solution such adaptation may include a conversion of ready-made libraries by ligation of special adapters “on top” of the original technical sequences for Illumina. Using this approach, only the optimization of the final stage is necessary. But, obviously, the “useful length” of reads is significantly limited in this case and, in addition, the library preparation protocol is lengthened along with the increased cost of the prepared library. These disadvantages make the conversion of ready-made libraries a suboptimal option for a clinically oriented NGS test.

To our knowledge, this study is the first to outline the successful adaptation of PCR-based library preparation for MGI, highlighting its ability to amplify and sequence the whole range of amplicons and demonstrating its high diagnostic efficiency. One of the key strengths of PCR-based library preparation is its ability to selectively amplify target regions of interest, thereby enriching the sample for variants of clinical relevance. This targeted approach minimizes sequencing costs and computational resources while maximizing the detection of actionable alterations. Moreover, the scalability of PCR-based library preparation makes it suitable for processing a large number of samples, facilitating high-throughput alteration screening in clinical settings. However, despite its advantages, the adaptation of PCR-based library preparation for MGI comes with its own set of challenges. Optimization of PCR conditions, including annealing temperature and amplification cycles as well as component composition of multiplex PCR mastermix, activator, ligase buffer, circularization kit and so on, – are crucial to ensure uniform coverage and minimize amplification biases.

Obtained data show the success of amplicon library adaptation to MGI platform in terms of diagnostic use. The integration of PCR-based library preparation with high-throughput MGI platform to sequence small amplicon panels allowed to achieve high coverage and high diagnostic efficiency thereof as compared to broad sequencing approach (Fig 3). We tested three library preparation protocol versions varied by key components: multiplex PCR mastermix for targeted enrichment, an activator for the preparation of amplicon sticky ends and an index PCR mastermix. None of the protocols tested demonstrated any bias of amplicon coverage depending on the amplicon length (Fig 5G-H) or padding amplicon nucleotide sequences (Fig 5A-D). Per-amplicon coverage of sticky-end libraries sequenced on Illumina and MGI were highly correlated (Fig 2C) demonstrating that the platform itself does not introduce any bias to amplicon coverage. At the same time some variations of protocols were characterized by low uniformity and high count of amplicon drop-out due to low coverage of GC-low and GC-high amplicons (Fig 5E-F). It remains unclear what is the reason for the GC distribution shift in amplicon data obtained from MGI. Obviously, it may be overcome by redesigning the oligo panel or by adjustment of PCR regimens but excessive coverage on MGI turns this problem into irrelevant from a diagnostic point of view. Moreover, low uniformity coming from GC-low and GC-high amplicons signifies the importance of correct component composition of library preparation kit, especially activator for ligation.

In conclusion, WES is still an option for genomic DNA sequencing in search of germline variants, however amplicon-based libraries are superior to it in tumor testing. Along with easy-to-use protocol and lower costs per sample, amplicon-based approach can lead DNBSEQ technique towards routine molecular pathology. In this work we provide a benchmark for a high-capacity sequencing platform applied to high-throughput cancer mutation testing in clinical practice. Continued innovation and collaboration in this field are essential to realizing the full potential of MGI platform for PCR-based library sequencing.
